# Supplementary material for: Bimanual Movement Characteristics and Real-World Performance Following Hand–Arm Bimanual Intensive Therapy in Children with Unilateral Cerebral Palsy
Source: Behav Sci (Basel). 2023 Aug 13;13(8):681. doi: 10.3390/bs13080681 (PMC10451828; doi:10.3390/bs13080681)
Supplement: Supplementary file 1 [file behavsci-13-00681-s001.zip › Supplementary Material 1.pdf]

Supplementary Material 1: Activities included in the HABIT protocol

Activity Category	Goal	Activities and games	Time	Type of involved hand use	Parameters manipulated
**Gross motor activities**	Repetitive task practice that involves shoulder, elbow, wrist, and hand activities in a functional context	Basketball, baseball, fishing, bowling, magnetic builders, badminton with air balloon, screwing nuts and bolts, Velcro tennis paddle, catch and throw, dart game with Velcro ball, ring toss, play dough / putti, activities in a play sand, construction toys	15 - 20 minutes for each activity	Stabilizer, manipulator, active/passive assist, symmetrical or asymmetrical movements, gross motor grasp, gross movements of the upper extremity	Spatial and temporal components, frequency, accuracy, the assisting hand use
**Fine motor activities**	Repetitive task practice for precision activities that involve fine motor grasps, in- hand manipulation, hand opening, release and finger isolation activities	Card games (flipping), opening and closing a lid of jar/bottle, paper tearing and tracing, beads and strings, keyboard activities, finger aerobics, scissor skills, sticks and beads, geometric ring set stacking, magnetic building sticks and blocks, Jenga, magnetic tiles, drawing, paper bag puppets, paper plate crafts, connect 4, LCD tablet.	15 - 20 minutes for each activity	Stabilizer, manipulator, active/passive assist, symmetrical or asymmetrical movements, reach, lift, grasp, release, finger isolation, supination, and pronation grasp	Spatial and temporal components, frequency, accuracy, the assisting hand use
**Strength activities**	Repetitive task practice for pinch and grasp activities that require hook, spherical, cylindrical grasps, tip to tip and pulp to pulp pinches (2 - 3 chuck) and prehensions	Squeezers, textured pull and stretch balls, suction toys, pulling balls from Velcro tennis paddle, Tug of war game, pull tubes, Thera putti, passing a football with both hands, weight cuffs, dumbbells, weighted bars.	15 - 20 minutes for each activity	Stabilizer, manipulator, active/ passive assist, symmetrical or asymmetrical movements, reach, grips, fingertip forces.	Spatial and temporal components, frequency, accuracy, the assisting hand use

Activity category	Goal	Activities and Games	Time	Type of involved hand use	Parameters manipulated
**Functional activities**	To train the child on functional activities that require bimanual coordination	Tying shoelaces, making a tie, kneading a cake and cookie dough, zipping jacket, buttoning a shirt, using a fork and knife, chopping vegetables, don and doff jacket, removing lids of different sized containers, folding clothes, opening doorknob, using lock and key, assembling nuts and bolts, fastening paperclips, swinging tennis and badminton rackets, swinging baseball bat	20 minutes	Stabilizer, manipulator, active/passive assist, symmetrical or asymmetrical movements, reach, lift, grasp, release, finger isolation, supination, and pronation grasp	Spatial and temporal components, frequency, accuracy, the assisting hand use
**Group activities**	Warm up, increase the interests of children, encourage bilateral use in play/ sport related tasks, to bring out competitive spirit and performance	Parachute, noodle hockey, hungry hippo, prone scooter race, Lego building competition, passing the football	30 minutes/ day	Upper extremity movements	
