# Supplementary material for: Bimanual Movement Characteristics and Real-World Performance Following Hand–Arm Bimanual Intensive Therapy in Children with Unilateral Cerebral Palsy
Source: Behav Sci (Basel). 2023 Aug 13;13(8):681. doi: 10.3390/bs13080681 (PMC10451828; doi:10.3390/bs13080681)
Supplement: Supplementary file 1 [file behavsci-13-00681-s001.zip › Supplementary Material 2.pdf]

Supplementary Material 2: Representative example of pre- and post-HABIT density plots of a child with unilateral cerebral palsy

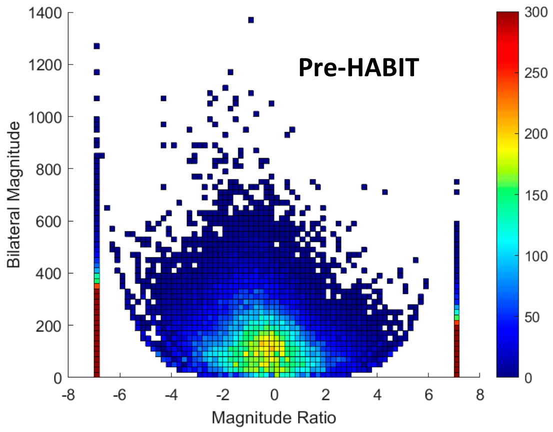

Figure S1 A

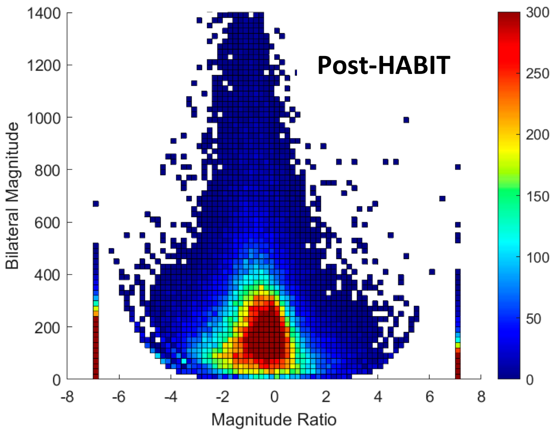

Figure S1 B

Accelerometer Variables	Pre-HABIT	Post-HABIT
Use Ratio	0.62	0.75
Magnitude Ratio	-4.90	-1.13
Bilateral Magnitude	67.90	108.61
Median Acceleration	1.00	24.35
Acceleration Variability	50.39	73.95

Table S1 shows the pre- and post-HABIT changes in the accelerometer derived variables following 30-hours HABIT in this representative participant data.

Supplementary Figures S1A,B show density plots displaying pre- and post-HABIT upper extremity use in daily life of an 11-year-old, male with right side hemiplegia. The data was acquired by wearing accelerometers on the affected and the less affected wrists and plotted on a second-by-second basis. The x-axis (magnitude ratio) indicates the contribution of each extremity to the task, and the y-axis indicates the overall intensity of movement. The right and left halves of the plots represent the affected and less affected upper extremities, respectively. The dots depict the counts/movements made by each extremity. The color represents frequency, with the large color bar scale on the right side of the figure, where brighter colors indicate greater frequencies. The small bars at -7 and 7 represent unilateral dominant and non-dominant activity, respectively.

Table S1 shows the pre- and post-HABIT changes in accelerometry derived variables. As compared to pre-HABIT, the post-HABIT density plot appears more symmetrical, as represented by a substantial increase in the use ratio (pre- and post-HABIT change= 0.13), which suggests more active use of the affected extremity post-HABIT. The change in use ratio is paralleled with increase in the number of movements, as represented by greater number of dots on the right half of the post-HABIT density plot (Figure S1B)

compared to pre-HABIT density plot (Figure S1A). The cool colors of the middle portion of the pre-HABIT (Figure S1A) plot compared to the bright colors in the post-HABIT (Figure S1B) plot reflect higher frequencies of movement following HABIT. The overall peak (representing bilateral magnitude) is much higher (pre- and post-HABIT change= 40.71) during the post-training relative to the pre-training, indicating higher intensities of activities. Overall, on closely observing both the density plots, we can infer that 30 hour of HABIT resulted in a significant change in the performance of the affected extremity in the real world as reflected by accelerometry.
