# Supplementary material for: Bimanual Movement Characteristics and Real-World Performance Following Hand–Arm Bimanual Intensive Therapy in Children with Unilateral Cerebral Palsy
Source: Behav Sci (Basel). 2023 Aug 13;13(8):681. doi: 10.3390/bs13080681 (PMC10451828; doi:10.3390/bs13080681)
Supplement: Supplementary file 1 [file behavsci-13-00681-s001.zip › Supplementary Material 3.pdf]

Supplementary Material 3: Profile plots illustrating individual differences in accelerometer-derived variables

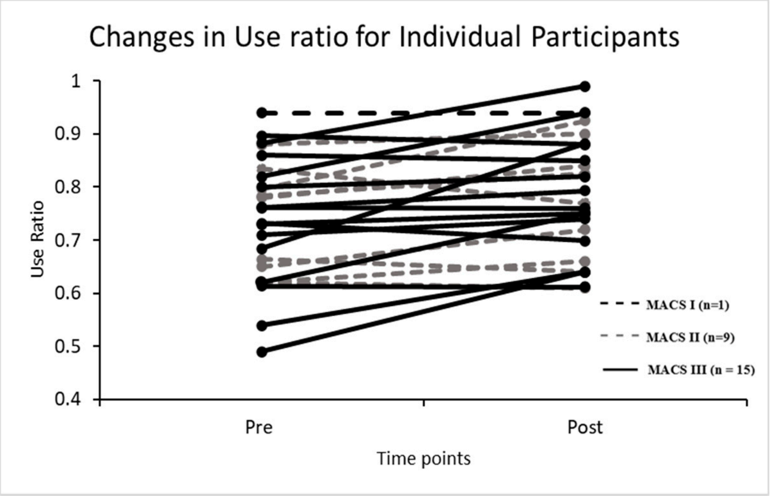

(a)

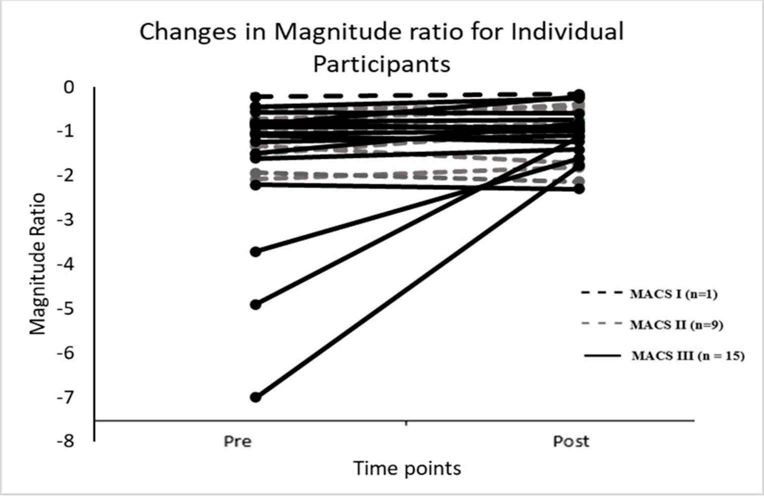

(b)

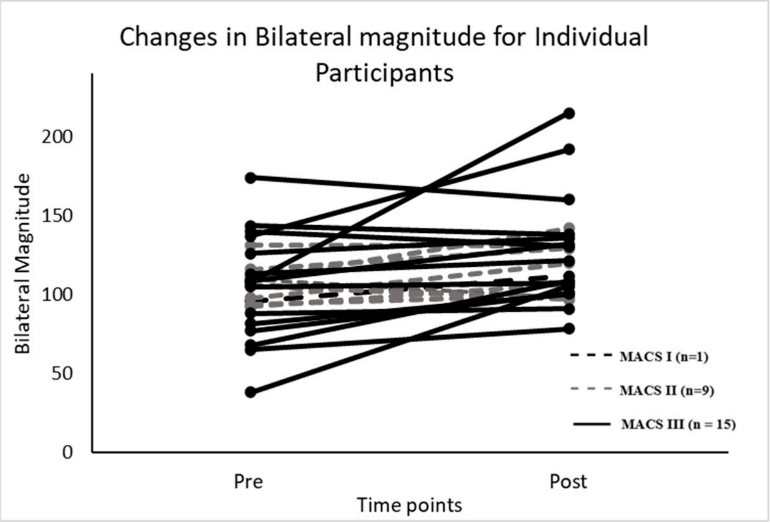

(c)

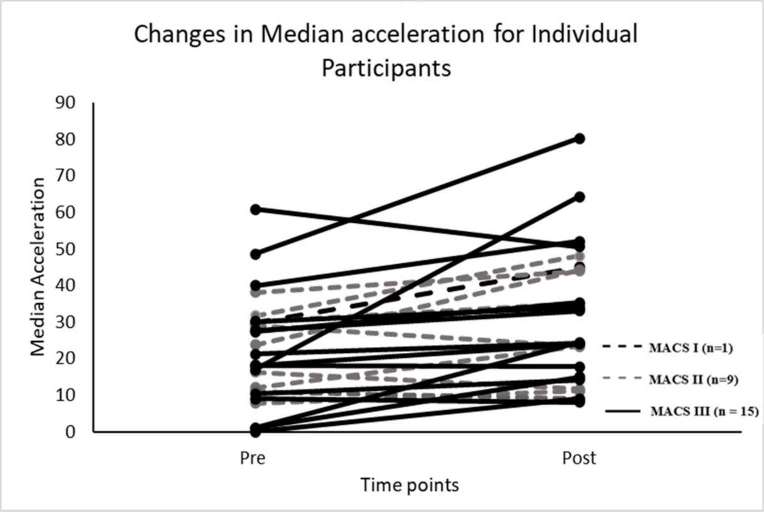

(d)

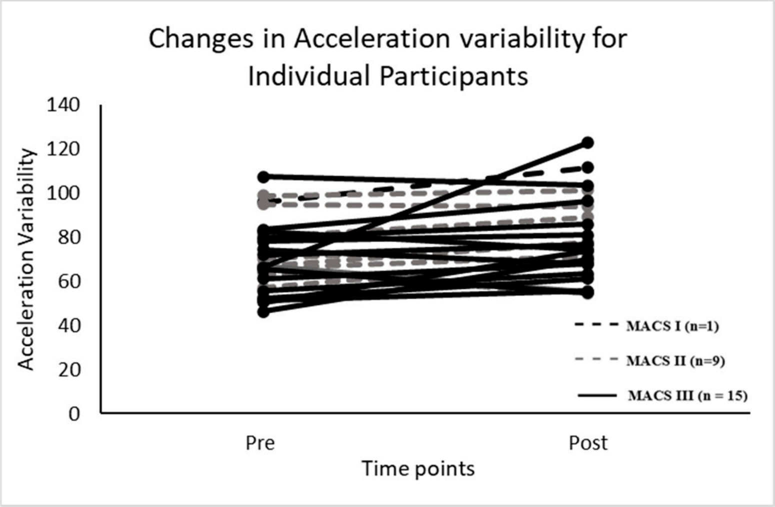

(e)

Supplementary Figure S2: Profile plots illustrate individual differences in accelerometer-derived variables, demonstrating the changes in performance before and after HABIT training. Participants are categorized according to the MACS levels (I - III), as indicated by the legends within the plots. "Pre" refers to the baseline assessment, while "post" refers to the assessment conducted within one week following HABIT. The values displayed at the pre- and post-time points represent the individual participant values recorded by the accelerometers. The variables depicted in the plots are as follows: (a) Use ratio, (b) Magnitude ratio, (c) Bilateral magnitude, (d) Median acceleration, and (e) Acceleration variability from pre- to post-HABIT.
